# Allergic rhinitis: Review of the diagnosis and management: South African Allergic Rhinitis Working Group

**DOI:** 10.4102/safp.v65i1.5806

**Published:** 2023-10-30

**Authors:** Guy A. Richards, Marinda McDonald, Claudia L. Gray, Pieter de Waal, Ray Friedman, Maurice Hockman, Sarah J. Karabus, Cornelia M. Lodder, Tshegofatso Mabelane, Sylvia M. Mosito, Ashen Nanan, Jonny G. Peter, Traugott H.C. Quitter, Riaz Seedat, Sylvia van den Berg, Andre van Niekerk, Eftyhia Vardas, Charles Feldman

**Affiliations:** 1 Department of Pulmonology, Faculty of Health Sciences, University of the Witwatersrand, Johannesburg, South Africa; 2 The Allergy Clinic, Blairgowrie, Johannesburg, South Africa; 3 Department of Paediatric Allergy, Faculty of Child Health, University of Cape Town, Cape Town, South Africa; 4 Department of Paediatric Allergy, Private Practice, Vincent Palotti Hospital, Cape Town, South Africa; 5 Department of Allergy, Pulmonology and Immunology, Faculty of Health Sciences, University of the Free State, Bloemfontein, South Africa; 6 Private Practice, Mediclinic Panorama, Cape Town, South Africa; 7 Department of Otorhinolaryngology, Private Practice, Mediclinic Sandton, Sandton, South Africa; 8 Johannesburg Cochlear Implant Programme, Netcare Linksfield Hospital, Johannesburg, South Africa; 9 Allergy Division, Faculty of Health Sciences, Red Cross War Memorial Children’s Hospital, University of Cape Town, Cape Town, South Africa; 10 Department of Allergy and Asthma Clinic, Private Practice, George, South Africa; 11 Department of Internal Medicine, Faculty of Health Sciences, Sefako Makgato Health Sciences University, Pretoria, South Africa; 12 Department of Otolaryngology, Faculty of Health Sciences, University of Pretoria, Pretoria, South Africa; 13 Department of Otorhinolaryngology, Faculty of Health Sciences, University of the Witwatersrand, Johannesburg, South Africa; 14 Department of Medicine, Faculty of Health sciences, University of Cape Town, Cape Town, South Africa; 15 Department of Otorhinolaryngology – Head and Neck Surgery, Faculty of Health Sciences, University of Pretoria, Pretoria, South Africa; 16 Department of Otorhinolaryngology, Faculty of Health Sciences, University of the Free State, Bloemfontein, South Africa; 17 Department of Immunology, Ampath Laboratories, Pretoria, South Africa; 18 Department of Paediatrics and Child Health, Faculty of Health Sciences, University of Pretoria, Pretoria, South Africa; 19 Departments of Inborn Errors of Immunity and Allergology, Faculty of Sciences, University of Pretoria, Pretoria, South Africa; 20 Department of Immunology, Faculty of Health Sciences, University of Pretoria, Pretoria, South Africa; 21 Department of Clinical Virology, Faculty of Health Sciences, University of Stellenbosch, Stellenbosch, South Africa; 22 Department of Virology Allergy and Immunology, Lancet Laboratories, Johannesburg, South Africa; 23 Department of Medicine, Faculty of Health Sciences, University of the Witwatersrand, Johannesburg, South Africa

**Keywords:** allergic rhinitis, intranasal corticosteroids, antihistamines, immunotherapy, saline rinse

## Abstract

**Background:**

Allergic rhinitis (AR) has a significant impact on the community as a whole with regard to quality of life and its relationship to allergic multi-morbidities. Appropriate diagnosis, treatment and review of the efficacy of interventions can ameliorate these effects. Yet, the importance of AR is often overlooked, and appropriate therapy is neglected. The availability of effective medications and knowledge as to management are often lacking in both public and private health systems.

**Methods:**

This review is based on a comprehensive literature search and detailed discussions by the South African Allergic Rhinitis Working Group (SAARWG).

**Results:**

The working group provided up-to-date recommendations on the epidemiology, pathology, diagnosis and management of AR, appropriate to the South African setting.

**Conclusion:**

Allergic rhinitis causes significant, often unappreciated, morbidity. It is a complex disease related to an inflammatory response to environmental allergens. Therapy involves education, evaluation of allergen sensitisation, pharmacological treatment, allergen immunotherapy (AIT) and evaluation of the success of interventions. Regular use of saline; the important role of intranasal corticosteroids, including those combined with topical antihistamines and reduction in the use of systemic steroids are key. Practitioners should have a thorough knowledge of associated morbidities and the need for specialist referral.

**Contribution:**

This review summarises the latest developments in the diagnosis and management of AR such that it is a resource that allows easy access for family practitioners and specialists alike.

## Introduction

Allergic rhinitis (AR) is one of the most common chronic conditions with a prevalence of 10% – 40%.^1,2,3,4,5,6,7,8^ It can cause significant discomfort and a marked reduction in productivity and quality of life (QoL).^8^ Moreover, its consequences can be serious, including contributing to asthma exacerbations and comorbidities such as rhinosinusitis and otitis media; increasing susceptibility to viral illnesses and impacting on taste, smell and sleep quality.^7,8,9,10^ Poor sleep quality can result in chronic fatigue, daytime sleepiness and learning problems in children.^11^ Allergic rhinitis can also aggravate mood disorders such as depression and decrease the ability to concentrate.^12,13^ Despite this, it is significantly underdiagnosed and sub-optimally treated, particularly in children where AR symptoms may be attributed to viral infections.^7^

Allergic rhinitis and asthma often coexist (united airway concept), and AR is a risk factor for the development of asthma.^14^ In patients with asthma, AR may be associated with poor control of the disease.^14,15^ Appropriate treatment of AR can result in a significant improvement in patients’ QoL, as well as improve the control of comorbid conditions such as asthma.^14,15^ This can reduce the overall cost of asthma treatment and reduce the number of patients with uncontrolled asthma requiring treatment at the hospital level.

Both the prevalence and consequences of AR have led the World Allergy Organization to label it ‘a global public health concern’.^16^ Excellent comprehensive guidelines are available^9,17,18,19,20,21^ but are generally written in, and for, high-income countries; whereas they are mostly universally applicable, there are local factors in South Africa (SA) that call for some unique recommendations, including:

- *Economic issues*: South Africa represents a resource-poor setting with priority given to infectious diseases and diseases considered to be more severe. Funds for medications for AR are frequently not a priority in the public health sector. However, untreated or poorly treated AR may have a greater economic cost as a result of absenteeism or reduced productivity.^22^
- *Practical issues*: Distance to hospital or clinic, single-parent households and inability to take time off work make clinic visits difficult.
- *Understanding of health-related issues*: This may be affected by poor health literacy and dominant traditional beliefs, negatively affecting compliance.

The aim of this consensus document, produced by the SAARWG, is to review up-to-date recommendations for AR applicable to SA.

## Epidemiology

Allergic rhinitis affects between 10% and 40% of children and adults worldwide,^1,2,3,4,5,6,7,8^ approximately 80% developing before the age of 20 years, with a peak at 20–40 years and then a gradual decline.^6,8^ The burden in low- and middle-income countries is similarly substantial and has been increasing since the 1990s.^1,2,6^ In the International Study of Asthma and Allergies in Childhood (ISAAC), the SA cohort of 13- to 14-year olds showed substantial and increasing prevalence from 30.4% in 1995 to 38.5% in 2002.^2^ Urbanisation and increasing levels of pollutants, as well as changes in pollen concentrations, allergenic potential and composition because of climate change, have been implicated in the increase in the prevalence of AR.^23,24^

## Pathophysiology

Allergic rhinitis is a result of a Type 1 hypersensitivity reaction of the nasal mucosa. Allergens deposited onto the nasal mucosa of sensitised individuals bind to allergen-specific immunoglobulin E (IgE) on the surface of mast cells, resulting in the release of preformed mediators such as histamine. This causes the early phase of the allergic response and leads to acute symptoms such as itching, sneezing and rhinorrhoea.^6,7,25^ The late phase of the allergic response, which precipitates a cycle of chronic allergic symptoms, manifests 4 h – 6 h after allergen exposure, with nasal mucosal inflammation from activation and influx of inflammatory cells, including T-cells, eosinophils, basophils and neutrophils.^6,25^

Priming (increased nasal responsiveness to an allergen with repeated allergen exposure) occurs as a result of increased numbers of mast cells in the epithelium, increased permeability of the epithelium and easier allergen penetration to IgE-bearing cells and exaggerated responses of the nasal end organs.^6,8^ Air pollutants can also contribute to priming. Treatment with intranasal corticosteroids (INCS) can suppress the priming response.^6,8,25^

## Clinical diagnosis

The diagnosis relies chiefly on clinical assessment and laboratory tests indicating allergic sensitisation. Clinical assessment should include a thorough history recording duration, seasonality and severity of symptoms, and examination.^7^ Nasal and non-nasal symptoms can occur.^7,25,26^

Symptoms of AR (which may be prolonged after allergen exposure) include the following:

*Nasal symptoms*:

- Rhinorrhoea (anterior and posterior), sneezing, nasal blockage and itching and hyperreactivity of the mucosa to other allergens and non-allergic stimuli (e.g., irritants and strong odours)

*Non-nasal associations*:

- Allergic conjunctivitis, palatal itching, cough from postnasal drip, asthma exacerbations, sinusitis or otitis media.
- An impact on QoL, specifically cognitive dysfunction and sleep disturbance.

### Examination

The examination should assess for signs of atopy such as the ‘allergic facies’ (pallor, allergic shiners, nasal creases, Dennie-Morgan lines and mouth breathing). The inferior turbinate should be examined for swelling and pallor. The patient should be evaluated for concomitant allergic diseases such as eczema and asthma. Comorbidities such as chronic rhinosinusitis (CRS), otitis media and hearing loss should be quantified. Other factors that can also cause nasal obstruction such as nasal polyposis, septal deviation, nasal deformities or mid-facial hypoplasia should be excluded.

### Imaging

Plain film sinus X-rays have no place in the diagnosis. Computed tomography scanning should be reserved for suspected chronic sinus disease, particularly where surgery is contemplated.

## Classification of severity

**FIGURE 1:**
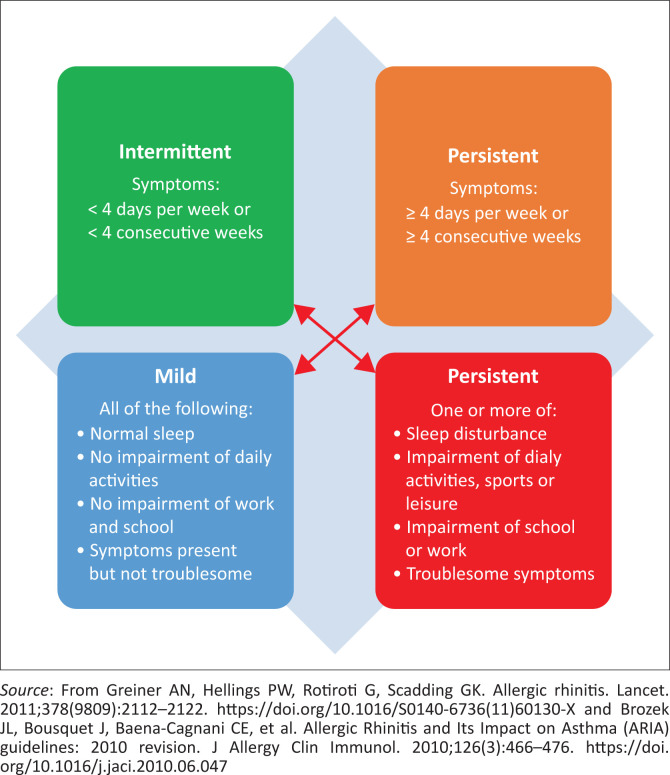
Allergic Rhinitis and Its Impact on Asthma guidelines on allergic rhinitis categorisation in untreated patients.

In SA, the Allergic Rhinitis and Its Impact on Asthma (ARIA) guidelines are widely applied^9,17^ despite the recognition of some weaknesses.^26,27^ These guidelines divide symptoms into intermittent or persistent and severity into mild or moderate-severe (Figure 1).^7,9^

## Differential diagnosis

The differential diagnoses of AR must be considered to plan for appropriate testing (Table 1).^6,7,8,25,28^

**TABLE 1:** Differential diagnosis of allergic rhinitis.

Inflammatory	Neurogenic/Vasomotor	Anatomical
Occupational rhinitis Chemical rhinitis Autoimmune, granulomatous and vascular rhinitis Fungal rhinosinusitis Non-steroidal exacerbated rhinitis/Samter’s triad Acute or recurrent infective rhinosinusitis Chronic rhinosinusitis with or without nasal polyposis Primary immune deficiency Crèche syndrome Cystic fibrosis	Vasomotor rhinitis Rhinitis medicamentosa Atrophic rhinitis Age-related rhinitis Smoke-induced rhinitis Non-allergic rhinitis with eosinophilia (NARES)	Deviated septum/septal spur Empty nose syndrome Antrochoanal polyp Foreign body Adenoidal hypertrophy Primary ciliary dyskinesia

*Source:* Please see the full reference list of the article Sin B, Togias A. Pathophysiology of allergic and non-allergic rhinitis. Proc Am Thorac Soc 2011;8(1):106–114. https://doi.org/10.1513/pats.201008-057RN, for more information

## Testing for allergen sensitisation

Objective laboratory testing can identify patients at increased risk for severe disease, direct preventative steps to minimise allergen exposure(s) and tailor treatment and allergen immunotherapy (AIT).^29^ However, indiscriminate and extensive specific IgE testing is not indicated where history and examination suggest AR.

Various recommendations regarding prevailing aero-allergens in South Africa have been made and subsequently modified.^29,30,31^ The 2015 iteration included the pooled specific IgE aeroallergen screen (Phadiatop^®^) for which there is an extensive evidence base to support its use as a single rule-out test.^32^

A panel of specific IgEs to aeroallergens deemed most relevant to SA was established in 2014 by the Allergic Rhinitis Diagnostic Working Group (ARDWG). This included indoor allergens (house dust mites (HDM) (*Dermatophagoides pteronissinus* [Der p] and *farinae* [Der f]), cat, dog, moulds (including *Alternaria, Epicoccum, Cladosporium*, and cockroach), and outdoor allergens (Rye grass, Bermuda grass). Since then, specific IgE testing over 3 years (November 2019 – October 2022) from two private laboratories revealed that 36.6% of Phadiatop^®^ tests were negative, confirming its usefulness as a screen-out tool and that testing analysis showed low positivity to the outdoor mould *Cladosporium* (m2).

**FIGURE 2:**
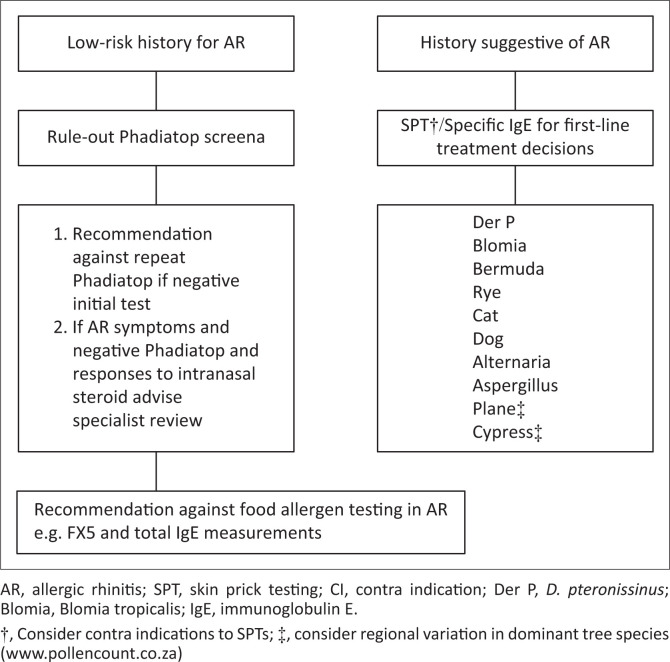
First-line testing recommendations to either rule out allergic rhinitis or guide first-line treatments or allergen avoidance strategies in allergic rhinitis.

On existing evidence, the following are recommended as first line for testing:

- Phadiatop^®^, although not specific for SA, is a cost-effective screen-out tool in patients with a history of possible AR.
- In patients with a history suggestive of AR, skin prick testing with the suggested ARDWG common allergens or specific IgE testing is indicated (Figure 2).
- If Phadiatop^®^ is positive, further analysis using the modified ARDWG panel may be performed to guide treatment.
- If Phadiatop^®^ is negative, an alternative diagnosis should be sought or local AR should be considered. Further allergy testing is only recommended if history suggests a specific aeroallergen trigger that has no cross reactivity with allergens in the Phadiatop^®^. Repeat Phadiatop^®^ testing is not recommended.
- *Cladosporium* has been removed from the ARDWG panel, and Plane and Cypress trees (Figure 2) included as early pollen monitoring data from across SA suggest these are the commonest allergenic tree pollens.^33^ However, pollen data are not yet comprehensive for all areas across SA, and other allergenic pollens (trees and weeds) may be relevant in certain areas^33^ (updated pollen data can be found at https://pollencount.co.za/).
- Specific tree panels should be considered based on clinical history, and where available, local sensitisation and pollen data (https://pollencount.co.za/).
- Food allergies very rarely cause AR, and hence, food allergy testing is generally discouraged.

## Management of allergic rhinitis in children and adults

Management rests on seven integrated pillars.^19^ They are:

- Education about AR and its therapy.
- Practical allergen avoidance and exposure reduction strategies.
- Nasal douching/irrigation and rinses.
- Pharmacological treatment:
  - Intranasal corticosteroids
  - Oral and intranasal antihistamines
  - Other (including leukotriene receptor antagonists [LTRA]).
- Patient evaluation for AIT.
- Measuring response to therapy.
- Patient evaluation for referral to a specialist.

### Education

Education is the cornerstone of effective management. Key points include explaining that AR is a chronic disease and that, apart from AIT, there is no curative treatment. Treatment options should be discussed with regard to cost, efficacy, ease of use and side-effect profile.

Shared decision-making is of utmost importance. One of the main goals of shared decision-making is long-term adherence to treatment,^34,35^ which is required for the successful treatment of AR.^36^ This is achieved through discussing the available therapeutic options and agreeing on a treatment plan that best serves the needs of the patient and which ensures compliance with and persistence with the plan.^34,35^

Daily medication for persistent symptoms or intermittent use for seasonal symptoms should be discussed, as should the correct dosage, frequency and time of dosing.

Poor treatment adherence is an important barrier to treatment success, and questionnaires, such as the Medication Adherence Report Scale, a validated 5-item tool that assesses adherence, can be used in routine clinical practice.^28^

**FIGURE 3:**
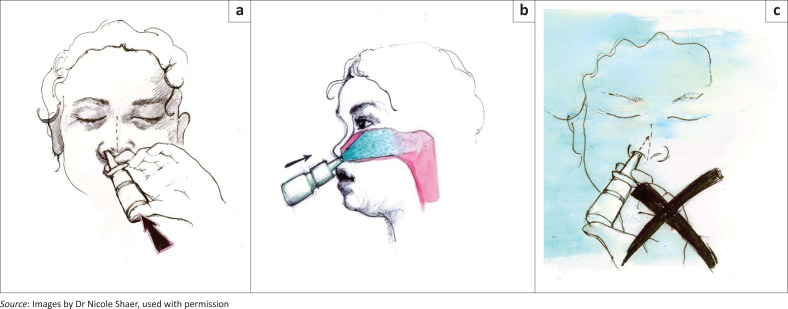
Technique for using nasal sprays. (a) Correct technique, (b) direction of spray using correct technique and (c) incorrect technique.

The correct method of using an INCS, by means of a physical demonstration, should be emphasised at the initial visit:

- After shaking and removing the lid, the nasal spray should be aimed towards the turbinates, which are on the lateral wall of the nasal passage.
- Ideally, the head should be tilted slightly forwards and, while closing the opposite nostril, one puff should be administered towards the outer side wall of the nose, aiming towards the ear and the back of the head, avoiding the nasal septum (Figure 3).
- Repeat the process in the other nostril.
- After each puff, the patient should try not to sniff, but rather pinch the nose between the thumb and index finger, holding the head neutrally forwards for a count of 10.
- Afterwards, the nose may be wiped, without blowing.

Written educational material containing this information may be helpful to cement the key points.

### Practical allergen avoidance and exposure reduction strategies

Allergen avoidance and environmental control measures aim to decrease exposure to aeroallergens and irritants to reduce the severity of symptoms (Table 2).^37,38,39,40^ The major outdoor allergens are pollens and fungal spores, while the major indoor allergens include HDM, pets, moulds and cockroaches. Avoidance measures can be cumbersome, expensive and not always practical, and hence, allergen sensitisation needs to be proven before advising on allergen reduction strategies.

**TABLE 2:** Environmental control and allergen avoidance measures.

Allergens	Control and measures
House dust mites	Use occlusive air-permeable fabric protectors for pillows, mattresses and duvets. Bedding should be washed in hot water and exposed to direct sunlight. Replace carpets with wooden floors or tiles. Loose carpets should be cleaned regularly and sun dried Vacuum with a vacuum cleaner with a high-efficiency. particulate air (HEPA) filter – adequate disposal of vacuum bag is important thereafter. Vacuum cleaning increases room dust, so a mask should be worn while vacuuming – leave the room for 20 min after vacuuming. Remove soft toys from the bedroom. Air conditioners are not advisable, as filters often contain house dust mite allergens, which may be recycled through rooms. Benzyl benzoate, tannic acid, acaricides and other anti-mite sprays have very little or unproven benefit. Humidifiers increase mould and HDM.
Cockroach	Do not leave food open overnight. Do not leave dishes in the sink overnight. Seal cracks and crevices. Vacuum or sweep the floor after every meal. The use of professional exterminators is advised. Use cockroach traps.
Pet allergens	Removal of pets only after proof of sensitisation with clinical symptoms directly related to pet exposure (patients and families are unlikely to adhere to this). Regular washing of pets. Keep pets out of the bedroom. Frequent vacuuming with vacuum cleaner equipped with a HEPA filter. Encase pillows and mattresses. There is no evidence that any breed of dog or cat is hypoallergenic.
Indoor moulds	Ensure adequate ventilation. Limit the number of indoor plants. Clean mould-infested surfaces with bleach. Repair of leaks. Removal of water-damaged materials. Run (exhaust) vents advisable in bathroom and kitchen. Regular vacuuming may reduce fungal spores, but replacing carpets with other types of flooring seems more effective.
Outdoor allergens (pollens and fungal spores)	Avoid outdoor activities and wear masks outdoors during peak pollen and mould periods by consulting pollen calendars and pollen forecasts/monitoring websites. Keep doors and windows closed. Change clothing when returning home. Mould-sensitive patients should avoid contact with decomposing leaves, grasses and grains.

*Source:* Please see the full reference list of the article Kalayci O, Miligkos M, Pozo Beltrán CF, et al. The role of environmental allergen control in the management of asthma. World Allergy Organ J. 2022;15(3):100634. https://doi.org/10.1016/j.waojou.2022.100634, for more information

Note: Further information on reduction of common allergens is available on the Allergy Foundation website – https://www.allergyfoundation.co.za/patient-information/en/allergens/. HDM, house dust mites.

### Nasal saline douches/irrigation and rinses

Nasal irrigation with hypertonic or isotonic saline is a simple, inexpensive and effective adjunct to therapy, by squirt, pump, gravity (e.g., ‘neti-pot’) or spray bottle systems. Nasal rinses remove allergens, irritants and inflammatory mediators and clear accumulated mucus, optimising mucociliary clearance. Saline rinsing is safe in children and adults and reduces disease severity and symptom scores.^41^

Whenever possible, saline irrigation should precede the administration of INCS to remove debris for better delivery of INCS.

In a resource-constrained environment, the following recipe can be used in place of commercially available products for nasal irrigation^42^:

- In a clean container, mix 3 teaspoons iodide-free salt with 1 teaspoon bicarbonate of soda.
- Add 1 teaspoon of this mixture to 250 mL of lukewarm distilled or boiled water.
- Using a soft ear bulb or a commercial device, draw up the solution, lean over a sink with the head held sideways and insert the mixture into the top nostril till it comes out of the bottom nostril.
- Then repeat on the opposite side.

### Pharmacological treatment of allergic rhinitis

**FIGURE 4:**
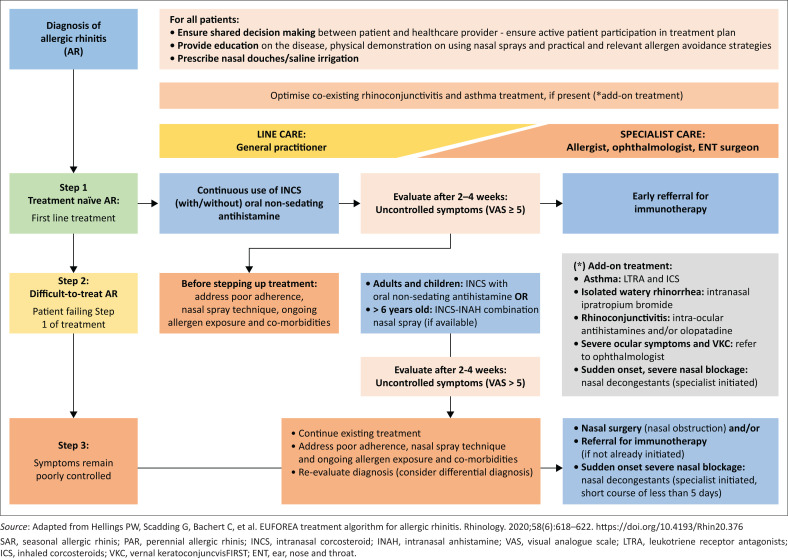
Flow diagram describing Management of Allergic Rhinitis.

The efficacy of the various classes of drugs for the treatment of AR is listed in Table 3^43,44^ and Figure 4.^19^

**TABLE 3:** Efficacy of various classes of drugs for allergic rhinitis.

Pharmacological agent	Nasal obstruction	Rhinorrhoea	Sneezing	Nasal itching
Intranasal corticosteroids	+++	+++	++	+
Antihistamines	+	+++	+++	+++
Combination intranasal corticosteroids and antihistamines	+++	+++	+++	+++
Intranasal cromones	+	+	+	+
Intranasal decongestants	++++	-	-	-
Anticholinergics	-	++	-	-
Leukotriene-receptor antagonists	++	+	-	-

*Source:* Adapted from Seedat RY. Treatment of allergic rhinitis. Curr Allergy Clin Immunol. 2013;26(1):11–16 and Sur DKC, Plesa ML. Treatment of allergic rhinitis. Allerg Rhinitis. 2015;92(11):985–992

#### Intranasal corticosteroids

Intranasal corticosteroids are the pharmacological treatment of choice for all forms of AR as they are effective against a wide range of symptoms.^17,19,25,29^ They are used intermittently for seasonal disease and continuously for perennial disease.^30^ Efficacy should be reviewed after 2–4 weeks in treatment-naïve patients. If still symptomatic, a combination of INCS and antihistamine is advised.

Side effects of INCS are mostly because of local irritation and include nasal dryness, a burning sensation inside the nose, blood-tinged nasal secretions and epistaxis.^21^ Hydrating the nose with saline may reduce these side effects. Erroneously aiming the spray towards the nasal septum is an important contributor to local side effects.

All newer INCS are safe and effective. However, these molecules have structural differences that influence glucocorticoid receptor-binding affinity and topical anti-inflammatory potency. These differences also alter the physicochemical properties such as solubility, lipophilicity and permeability, which in turn influence the pharmacokinetic properties and the systemic activity and therapeutic index.^45,46^ Molecules such as fluticasone propionate, fluticasone furoate, ciclesonide and mometasone furoate have increased glucocorticoid receptor selectivity and binding affinity and greater uptake and retention in the nasal tissue and have negligible systemic bioavailability (< 1%) compared to molecules such as budesonide, beclomethasone dipropionate and triamcinolone.^46,47^

Intranasal corticosteroids decrease the release of inflammatory mediators and cytokines from inflammatory cells and provide effective symptomatic relief when used continuously or as needed. They are most effective when used regularly, or at least in prolonged ‘blocks’ of treatment, as the onset of action is 7 h to 12 h, with maximum benefit after 2 weeks of regular use. Intranasal corticosteroids with increased topical potency do not necessarily offer a therapeutic advantage relative to those with less potency.^47,48,49,50^

Intranasal corticosteroids are less likely to cause systemic side effects (e.g., adrenal suppression, bone fractures, growth suppression and ocular side effects) compared to oral and inhaled corticosteroids because of the lower dose and lower bioavailability.^48^ Care should still be taken when multiple different steroid formulations (e.g., topical, inhaled and intranasal) are used.

Short-term use of INCS drops can be considered for severe congestion (1–2 weeks), but long-term use of INCS drops, as opposed to nasal sprays, is strongly discouraged, as these have higher systemic bioavailability and are significantly more likely to cause systemic side effects.

Depot intramuscular steroid injections should not be used for the treatment of AR.^9,51^ Complications associated with their use include hypothalamic–pituitary–adrenal‑axis (HPA) suppression, hyperglycaemia, osteoporosis, avascular necrosis of the femoral head and gluteal subcutaneous atrophy.^51^

#### Systemic antihistamines

H1-AH dampen the effects of histamine during the early and late phase of allergic reactions. They are effective against itching, sneezing and rhinorrhoea but have little efficacy against congestion.

H^1^-AH are functionally classified as first- or newer- (second and third) generation AH. Third-generation formulations (e.g., desloratadine, levocetirizine and fexofenadine) are metabolites or enantiomers of second-generation AH and are theoretically safer and more efficacious than second-generation types.^52^

First-generation AH have poor receptor selectivity (also acts on serotonergic, cholinergic, α-adrenergic receptors, also act on cardiac potassium ion channels)^53^ and high lipid solubility, causing significant blood–brain barrier transgression. Because of the non-selectivity of receptor binding and propensity to side effects (including cardiac and gastrointestinal side effects, sedation, dry mouth, blurred vision),^52,53^ SAARWG strongly discourages the use of first-generation AH in the routine management of AR.^30^ Second- and third-generation AH are less sedating than the first-generation AH because of reduced brain H1 receptor occupancy.^54^ Fexofenadine does not cross the blood–brain barrier. Rupatidine is a platelet-activating factor antagonist in addition to its antihistaminic properties.^55^ In a systematic review of 45 randomised controlled trials, second-generation AH use in children was generally safe; however, some may cause sedation in certain patients.^56^

The SAARWG recommends the *exclusive use of newer generation AH* for AR treatment, with careful selection based on each patient’s unique profile. If side effects occur, a different, non-sedating AH may be tried; however, the EUFOREA guidelines on AR in children discourage AH switching, and an intranasal AH or INCS is preferred.^57^

#### Intranasal antihistamines

Topical INAH act rapidly (within 15 min) and have proven to be more effective than oral AH in the control of AR.^58^ They are effective and safe in children with AR.^17,57,58^ The major side effect is a bitter taste in the mouth, which is less with olopatadine than azelastine.^57^ However, INAH (e.g., olopatadine and azelastine) are costly and not readily available in SA. In 2021, a combination intranasal spray, mometasone/olopatadine became available in SA, with approval for use in teenagers and adults.

#### Topical nasal decongestants

Nasal decongestants contain phenylephrine, oxymetazoline and xylometazoline that cause vasoconstriction of the nasal mucosa when applied topically, increasing airflow and relieving congestion. However, they have no effects on the other symptoms of AR and may worsen rhinorrhoea. Use for more than 5 to 10 consecutive days can cause rhinitis medicamentosa (rebound congestion). They should be used only for a short period when nasal congestion is dominant and always with an INCS.^59^

#### Leukotriene receptor antagonists

Leukotriene receptor antagonists are similar in efficacy to oral AH but are more effective in improving night-time than daytime nasal symptoms than AH.^59^ They are less effective than INCS in improving overall symptoms and QoL and should not be used as first-line treatment.^25^ Combinations of AH and LTRA are discouraged because of cost, unless concomitant asthma (especially exercise-induced and/or aspirin-exacerbated respiratory disease) is present.^17^ In this case, an LTRA (rather than an oral AH) should be considered as an add-on to INCS, together with guideline-directed asthma treatment.^60^ A summary of South African Allergic Rhinitis Working Group – recommended practice points for the pharmacological treatment of allergic rhinitis is available in Table 4.^60^

**TABLE 4:** Summary of South African Allergic Rhinitis Working Group -recommended practice points for the pharmacological treatment of allergic rhinitis.

No.	Recommended practice points
1.	INCS are considered the first-line treatment for AR and are effective for all the nasal symptoms of AR.
2.	Maximal effect of INCS on symptom relief occurs only after 2 weeks of continuous use.
*3.*	*The use of saline sprays or rinses is effective to remove mucus, allergens and inflammatory cells, and regular use, preceding the use of INCS, should be encouraged.*
*4.*	*For moderate to severe allergic rhinitis, a combination INCS-local AH spray should be considered early in the treatment ladder, if available.*
5.	Newer-generation oral AH should be used as add-on therapy to INCS, if necessary.
6.	No newer-generation oral AH have been consistently shown to be more efficacious than another.
7.	AH address pruritus, sneezing and rhinorrhoea but, as opposed to the INCS, have minimal effects on nasal congestion.
8.	Newer generation oral AH can be used as monotherapy (first line), but only if a patient is reluctant to use INCS
9.	The choice of newer generation AH should be individualised, considering issues such as pregnancy, drug interactions and cost.
10.	Some non-sedating (newer generation) oral AH are registered for use in children aged 6 months and older (and are often prescribed ‘off-label’), while most other AH are registered for safe use in children older than 2 years of age.^61,62^
*11.*	*Older generation AH (alone or in combination with systemic corticosteroids or systemic decongestants) should not be used to treat viral infections.*
12.	AH do not prevent asthma in children with AR and/or eczema.
13.	In children (< 6 years of age), faster drug elimination may require twice daily instead of daily dosing (e.g., cetirizine and levocetirizine).
*14.*	*Currently, no data have been published on the ‘development of tolerance’ to AH, and no scientific evidence exists that encourages patients to rotate through different AH after a certain period of using a specific drug.*
15.	Because of unnecessary medication costs involved in dual therapy, the SAARWG, in line with the ARIA guideline, discourages the use of leukotriene receptor antagonists (LTRA) combined with oral AH in AR, unless concomitant asthma (especially exercise-induced and/or aspirin-exacerbated respiratory disease) is present. If this is the case, an LTRA (rather than an oral AH) is the drug of choice as add-on with INCS.^17^

*Source:* From Gray CL, Davis M, Friedman R, et al. The diagnosis and management of allergic rhinitis: Summary of recommendations by the South African Allergic Rhinitis Working Group (SAARWG) 2015. Curr Allergy Clin Immunol. 2015;28(4):285–295

AR, allergic rhinitis; ARIA, Allergic Rhinitis and Its Impact on Asthma; INCS, intranasal corticosteroids.

### Allergen immunotherapy

Allergen immunotherapy is a desensitisation process for IgE-mediated hypersensitivity to common allergens such as pollens, HDM and insect venoms. It is the only disease-modifying treatment available for AR.^17^

The administration of high-dose allergen, using sublingual (SLIT) or subcutaneous (SCIT) immunotherapy, suppresses the pro-allergic dendritic cell phenotype by inducing T-cell differentiation to regulatory phenotypes (Tregs). Induction of B regulatory cells to stimulate blocking antibodies further reduces mast cell degranulation.^63^

Allergen immunotherapy improves short- and long-term symptom severity, decreasing the need for medication for AR and protecting against the progression from AR to asthma.^64,65,66^ Allergen immunotherapy studies have further demonstrated a reduced need for asthma medications^67^ and a reduction in new aeroallergen sensitisations.^65^

Subcutaneous and SLIT are both effective once the causative allergen has been accurately identified by history and allergen sensitisation tests. Allergen provocation tests might be necessary in cases of high suspicion and inconclusive allergy test results.^57,68^

According to the ARIA-EAACI care pathway, both monosensitised (single dominant antigen) and polysensitised (multiple antigens) patients can benefit.^57,68^

Patients who should be considered for AIT include the following:

- Those in whom symptom control is not achieved with pharmacotherapy and allergen avoidance.
- Those in whom high medication doses with potential side effects are required, particularly corticosteroids.
- Those in whom adverse events have occurred on normal doses of pharmacotherapy.
- Those who would prefer not to have to take pharmacotherapy for prolonged periods.
- Children in whom AIT would potentially be a modifying intervention to prevent further sensitisations and to reduce the chance of developing asthma.
- Potentially adolescents or adults with pollen-food syndrome.

The duration of treatment should be for at least 3 years but needs to be individualised and might need to be continued for up to 5 years according to symptom severity and control.^69^

Asthma should be well controlled, and practitioners should be well versed in the management of adverse events, including rare cases of anaphylaxis.^70,71^

Currently, acquisition of AIT is made difficult by the fact that it is an expensive, unlicensed product with a single distributor in South Africa. For greater access, it would be preferable for products to be registered with the SA Health Products Regulatory Authority (SAHPRA). The acquisition cost of AIT is high, but the reduction in morbidity and medication costs makes it cost-effective.

### Response to treatment

Visual analogue scales (VAS) (visual aids 100 mm long with descriptors of severity on opposite ends) or AR control tests are increasingly used to evaluate control and treatment response, to detect adverse effects and to gauge the need for treatment adjustment in a reproducible manner.^72^ Tests of control should be validated and quick and easy to perform in routine clinical practice.^73^ Validated control tests include the ‘Control of Allergic Rhinitis and Asthma Test’ (CARAT), ‘Rhinitis Control Assessment Test’ (RCAT), ‘Allergic Rhinitis Control Test’ (ARCT) and ‘Sinonasal Outcome Test’ (SNOT) for CRS.^8,28^ The RQLQ questionnaire has been translated into Afrikaans, isiXhosa and isiZulu.^74^ The clinician should use the same control test consistently and regularly to monitor the AR.

### Indications for referral

Patients with AR can be successfully initiated on AR treatment by general practitioners. Treatment success should be evaluated 2–4 weeks after the initiation of therapy.

The following are indications for referral to a specialist (e.g., allergologist, ear, nose and throat [ENT] surgeon or ophthalmologist, according to symptoms):

- Poor or no response to treatment (based on VAS assessment).
- Need for initiation of AIT.
- Assessment of aeroallergen sensitisation if not available at the primary health care level.
- Atypical nasal symptoms and signs, including unilateral involvement, epistaxis and anosmia.
- The presence of nasal polyps, septal perforation, facial deformities and significant cervical lymphadenopathy.
- Severe co-morbid allergic diseases (e.g., atopic dermatitis, asthma and food allergy).
- Warning symptoms and signs of a possible underlying immune deficiency (e.g., cystic fibrosis, inborn errors of immunity and primary ciliary dyskinesia).
- The presence of severe ocular involvement (e.g., vernal keratoconjunctivitis).

## Indications for surgery in allergic rhinitis

Surgery for ‘pure’ AR is rarely needed but may be needed in severe cases to improve airflow (inferior turbinate surgery, adenoidectomy, septoplasty and polypectomy)^75,76^; to improve access to topical medications and to decrease disease burden before other procedures such as AIT, aspirin desensitisation or initiation of biological therapies.^77,78,79^

Surgery may be needed to manage complications of AR such as chronic or recurrent otitis media or for overlapping chronic conditions involving the nose and sinuses such as CRS with and without nasal polyps, non-steroidal (aspirin) exacerbated respiratory disease, cystic fibrosis, eosinophilic granulomatous polyangiitis (Churg-Strauss syndrome) and allergic fungal rhinosinusitis.^18^ Such patients should be referred for appropriate procedures by a surgeon skilled in rhinology and base of skull surgery.

## Multi-morbidities

Multi-morbidity is defined as the presence of one or more additional disorders co-occurring with a primary disorder. Multi-morbidities associated with AR include the following:

- Allergic disorder spectrum: asthma, atopic dermatitis, food allergy, eosinophilic oesophagitis, allergic conjunctivitis and anaphylaxis.
- Disorders of the upper airway, middle ear and Eustachian tube disease, sinusitis, turbinate and adenoid hypertrophy and pharyngeal and laryngeal disorders.
- Sleep disorders with secondary effects on concentration, behaviour and mood.

Treatment of AR will often result in an improvement of these associated multi-morbidities.^80^

## Conclusion

Allergic rhinitis causes significant, often unappreciated, morbidity in the community. It is a complex disease related to an inflammatory response to environmental allergens. Therapy involves education, evaluation of allergen sensitisation, pharmacological treatment, AIT and evaluation of the success of interventions. Regular use of saline, the important role of INCS, including those combined with topical antihistamines and the reduction in the use of systemic steroids are key. Practitioners should have a thorough knowledge of associated morbidities and the need for specialist referral.
