# Erratum: Allergic rhinitis: Review of the diagnosis and management: South African Allergic Rhinitis Working Group

**DOI:** 10.4102/safp.v67i1.6150

**Published:** 2025-03-17

**Authors:** Guy A. Richards, Marinda McDonald, Claudia L. Gray, Pieter de Waal, Ray Friedman, Maurice Hockman, Sarah J. Karabus, Cornelia M. Lodder, Tshegofatso Mabelane, Sylvia M. Mosito, Ashen Nanan, Jonny G. Peter, Traugott H.C. Quitter, Riaz Seedat, Sylvia van den Berg, Andre van Niekerk, Eftyhia Vardas, Charles Feldman

**Affiliations:** 1 Department of Pulmonology, Faculty of Health Sciences, University of the Witwatersrand, Johannesburg, South Africa; 2 The Allergy Clinic, Blairgowrie, Johannesburg, South Africa; 3 Department of Paediatric Allergy, Faculty of Child Health, University of Cape Town, Cape Town, South Africa; 4 Department of Paediatric Allergy, Private Practice, Vincent Palotti Hospital, Cape Town, South Africa; 5 Department of Allergy, Pulmonology and Immunology, Faculty of Health Sciences, University of the Free State, Bloemfontein, South Africa; 6 Private Practice, Mediclinic Panorama, Cape Town, South Africa; 7 Department of Otorhinolaryngology, Private Practice, Mediclinic Sandton, Sandton, South Africa; 8 Johannesburg Cochlear Implant Programme, Netcare Linksfield Hospital, Johannesburg, South Africa; 9 Allergy Division, Faculty of Health Sciences, Red Cross War Memorial Children’s Hospital, University of Cape Town, Cape Town, South Africa; 10 Department of Allergy and Asthma Clinic, Private Practice, George, South Africa; 11 Department of Internal Medicine, Faculty of Health Sciences, Sefako Makgato Health Sciences University, Pretoria, South Africa; 12 Department of Otolaryngology, Faculty of Health Sciences, University of Pretoria, Pretoria, South Africa; 13 Department of Otorhinolaryngology, Faculty of Health Sciences, University of the Witwatersrand, Johannesburg, South Africa; 14 Department of Medicine, Faculty of Health Sciences, University of Cape Town, Cape Town, South Africa; 15 Department of Otorhinolaryngology – Head and Neck Surgery, Faculty of Health Sciences, University of Pretoria, Pretoria, South Africa; 16 Department of Otorhinolaryngology, Faculty of Health Sciences, University of the Free State, Bloemfontein, South Africa; 17 Department of Immunology, Ampath Laboratories, Pretoria, South Africa; 18 Department of Paediatrics and Child Health, Faculty of Health Sciences, University of Pretoria, Pretoria, South Africa; 19 Departments of Inborn Errors of Immunity and Allergology, Faculty of Sciences, University of Pretoria, Pretoria, South Africa; 20 Department of Immunology, Faculty of Health Sciences, University of Pretoria, Pretoria, South Africa; 21 Department of Clinical Virology, Faculty of Health Sciences, University of Stellenbosch, Stellenbosch, South Africa; 22 Department of Virology Allergy and Immunology, Lancet Laboratories, Johannesburg, South Africa; 23 Department of Medicine, Faculty of Health Sciences, University of the Witwatersrand, Johannesburg, South Africa

In the published article, S Afr Fam Pract (2004). 2023;65(1), a5806. https://doi.org/10.4102/safp.v65i1.5806, there was an error in Figure 1, Allergic rhinitis and its impact on asthma guidelines on allergic rhinitis categorisation in untreated patients.

A heading in Figure 1 shows the word Persistent, which is incorrect. The heading should read Moderate to Severe.

The publisher apologises for this error. The correction does not change the study’s findings of significance or overall interpretation of the study’s results or the scientific conclusions of the article in any way.

